# Amido-Functionalized Magnetic Metal−Organic Frameworks Adsorbent for the Removal of Bisphenol A and Tetracycline

**DOI:** 10.3389/fchem.2021.707559

**Published:** 2021-08-06

**Authors:** Guangpu Zhang, Rong Wo, Zhe Sun, Lei Xiao, Guigao Liu, Gazi Hao, Hu Guo, Wei Jiang

**Affiliations:** National Special Superfine Powder Engineering Research Center of China, School of Chemistry and Chemical Engineering, Nanjing University of Science and Technology, Nanjing, China

**Keywords:** magnetic metal-organic framework, adsorption, kinetic, bisphenol A, tetracycline

## Abstract

In this paper, amido-functionalized MOFs with core/shell magnetic particles (Fe_3_O_4_@MIL-53(Al)-NH_2_) was prepared by the solvothermal method and characterized by X-ray diffraction (XRD), transmission electron microscopy (TEM), scanning electron microscopy (SEM), Fourier transform infrared (FT-IR), Vibrating Sample Magnetometer (VSM) and UV/VIS spectrophotometer. The influence of different factors on the adsorption effect of the pollutant, including adsorbent amounts, adsorption time, ionic strength and pH, were explored. It was found that the amine-decorated Fe_3_O_4_@MIL-53(Al)-NH_2_ were efficient for removal of contaminant, with the adsorption capacity for bisphenol A (234.1 mg/g) and tetracycline (84.8 mg/g) under the optimized conditions. The adsorption kinetics and the equilibrium adsorption data indicated that the adsorption process of BPA and TC was more compatible with the pseudo-second-order kinetic model and the Langmuir model, respectively. The thermodynamic values show the adsorption of the mentioned contaminant was spontaneous and endothermic. Moreover, the Fe_3_O_4_@MIL-53(Al)-NH_2_ adsorbent had good regeneration and reusability capacity after five cyclic utilization. All these results show Fe_3_O_4_@MIL-53(Al)-NH_2_ adsorbent could be a potential candidate for future water purification.

## Introduction

Given the serious problem of water pollution, increasingly growing number of researchers have devoted themselves to water treatment technology (Gleick, 2000). Metal–organic framework materials (MOFs) have a high specific surface area and developed pores, which are considered effective for sewage treatment (Joseph et al., 2019; Wang et al., 2019). MOFs are generally more efficient for sewage treatment compared with traditional adsorbents, such as activated carbon, zeolite, silica microspheres, and natural fibers (Ke et al., 2011; Patil et al., 2011). Haque et al. (2010) compared the adsorptive effects of MOF-235 and activated carbon for methyl orange and methylene blue in wastewater. The maximum adsorption capacity of MOF-235 on methyl orange was 477 mg/g, which was 43 times that of activated carbon, and that for methylene blue was 187 mg/g, which was 7 times that of activated carbon (AC). Park et al. (2013) compared the adsorption capacities of MIL-53(Cr), activated carbon (AC), and ultra–stable Y-zeolite (USY) for bisphenol A (BPA). Adsorption of BPA reached its maximum on MIL-53(Cr) (421 mg/g). The order of the adsorbent, ranked by their adsorption capacity in the descending order, was MIL-53(Cr)>AC > USY. This result is attributable to the п–п conjugation and hydrogen bonding between the benzene ring in BPA and the benzene ring in MIL-101-CR. Therefore, MOFs adsorbents have adsorption capacity superior to traditional materials, exhibiting enormous potential for effluent treatment.

Modification of MOFs often results in enhanced performance owing to functional groups. Well-established methods can be classified into functional group introduction (Lee et al., 2019), metal ion doping (Yang et al., 2019), binding with magnetic microspheres (Ke et al., 2013), polymer modification (Liu et al., 2019), and carbon material loading ^[^ (Liu et al., 2011; Zhao et al., 2011; Zlotea et al., 2011; Jeong et al., 2013; Li et al., 2013). The most commonly used modified functional group in MOFs is the amino group, which can adjust the channel structure (Halis et al., 2015), improve the stability of adsorbents in water, hydrogen bonding (Li et al., 2019), and electron transfer capabilities for target analytes. Amination methods are mainly categorized into pre-synthesis and post-synthesis modification. Liang et al. (2015) used aminated MOFs (MIL-68(In)-NH_2_) for the first time to realize the photocatalytic reduction of Cr (VI) into Cr (III) under visible– light irradiation, proving the great application prospect of aminated MOFs. P. Serra-Crespo group (Serra-Crespo et al., 2015) studied MIL-53(Al)-NH_2_ adsorbent for the separation of carbon dioxide from methane mixtures. They established a flexible adsorbent model using the Langmuir and the Freundlich model; MIL-53(Al)-NH_2_ exhibited higher adsorption and separation capacity than those of MIL-53(Al). Compared with non-aminated UIO-66, it is found that UIO-66-NH_2_ has higher selective adsorption capacity to 2-methyl-4-chlorophenoxyacetic acid (Wei et al., 2016), diclofenac sodium (Hasan et al., 2015), methylene blue, and methyl orange (Chen et al., 2015). Because of the superior performance of the aminated MOFs, modifying the material to endow the amino functional group will be beneficial to improve the performance and application value of the material.

Although the preparation of magnetic MOF (Fe_3_O_4_@MIL-53(Al)) has been reported by vortex-assisted dispersive magnetic solid phase extraction method (Boontongto and Burakham, 2020), which exhibits excellent combination of magnetite and MOFs for extraction of ten phenols, however, the factor on adsorption capacity, thermodynamic and kinetic characteristics of absorption for other vital organic pollutants have not been investigated. In the current work, we used the magnetic composite adsorbent Fe_3_O_4_@MIL-53(Al) with 2-aminopyterate (BDC-NH_2_) instead of 1,4-terphenzene as an organic ligand, to prepare the amido-MOFs with core/shell magnetic particles (Fe_3_O_4_@MIL-53(Al)-NH_2_) by solvothermal synthesis. With magnetic particles, adsorbents can be separated by an external magnetic field (Wang et al., 2014; Zheng et al., 2014; Pang et al., 2015; Gutierrez et al., 2017). Magnetic MOFs were subjected to can be carried out to structural modification or functional group modification to enhance the adsorption capacity or chemical stability of magnetic metal–organic skeleton composite particles for target pollutants. The effects of different factors on adsorption were evaluated using Fe_3_O_4_@MIL-53(Al)-NH_2_ for BPA and tetracycline adsorption. The adsorption mechanism of the adsorption was also determined, and the ability of the target pollutant to circulate was determined using the adsorption model. Under optimal adsorption conditions, the maximum adsorption capacity of Fe_3_O_4_@MIL-53(Al)-NH_2_ reached 234.1 mg/g for BPA and 84.8 mg/g for tetracycline, indicating its potential as an adsorbent for contaminant removal.

## Experimental Section

### Materials

Ferric chloride (FeCl_3_·6H_2_O), ethylene glycol, sodium acetate anhydrous (NaAc), *N*, *N*-dimethylformamide (DMF), methyl alcohol, sodium hydroxide, and hydrochloric acid were supplied by Sinopharm Chemical Reagent Co., Ltd., China. 2-Aminoterephthalic acid was purchased from Shanghai Dibai Biotechnology Co., Ltd. Trisodium citrate dihydrate and aluminum nitrate nonahydrate were provided by Xilong Scientific Co., Ltd., China. 1,4-Dicarboxybenzene was purchased from Nanjing Wanqing Pharm. Co., Ltd., China. BPA was supplied by Shanghai Aladdin BioChem Technology Co., Ltd., China, and tetracycline was provided by Nanjing Jiaozi Rattan Scientific Instrument Co., Ltd., China. Congo red, methylene blue and sodium chloride (NaCl) were purchased from Chengdu Kelong Chemical Industry, China. Absolute ethyl alcohol was supplied by Nanjing Chemical Reagent Co., Ltd., China. All reagents and solvents were used as received without further purification.

### Characterization

The chemical composition of Fe_3_O_4_@MIL-53(Al)-NH_2_ composite were determined by X-ray diffractometer (XRD) (D8 Advance, Bruker, Germany), using Cu K_α_ radiation in the range of 5–80° (2θ), voltage of 40 kV, current of 40 mA and wavelength of 1.54 Å. The Fourier transform infrared (FT-IR) spectroscopy were recorded in the solid state (KBr pellet method) by using Vector 22 (Bruker). Scanning electron microscope (SEM) images were obtained using a Model-S480 II FESEM. The size and morphology of the particles were also analyzed by TEM (Tecnai 12). The magnetic saturation strength of the sample was measured by VSM using the LakeShore 735 device. The test magnetic moment ranged from -20 kOe to 20 KOe. The specific surface areas of the different samples were determined by specific surface area testing (ASAP 2020). The testing voltage and current were 220 V and 50 Hz, respectively. The dye concentrations in the solution were determined by UV/vis spectrophotometry (Agilent Cary 100 UV-2600, Agilent). The spectral range was 200–800 nm. The maximum wavelengths of BPA and TC were 278 and 380 nm, respectively.

### Preparation of Fe_3_O_4_@MIL-53(Al)-NH_2_

**SCHEME 1:**
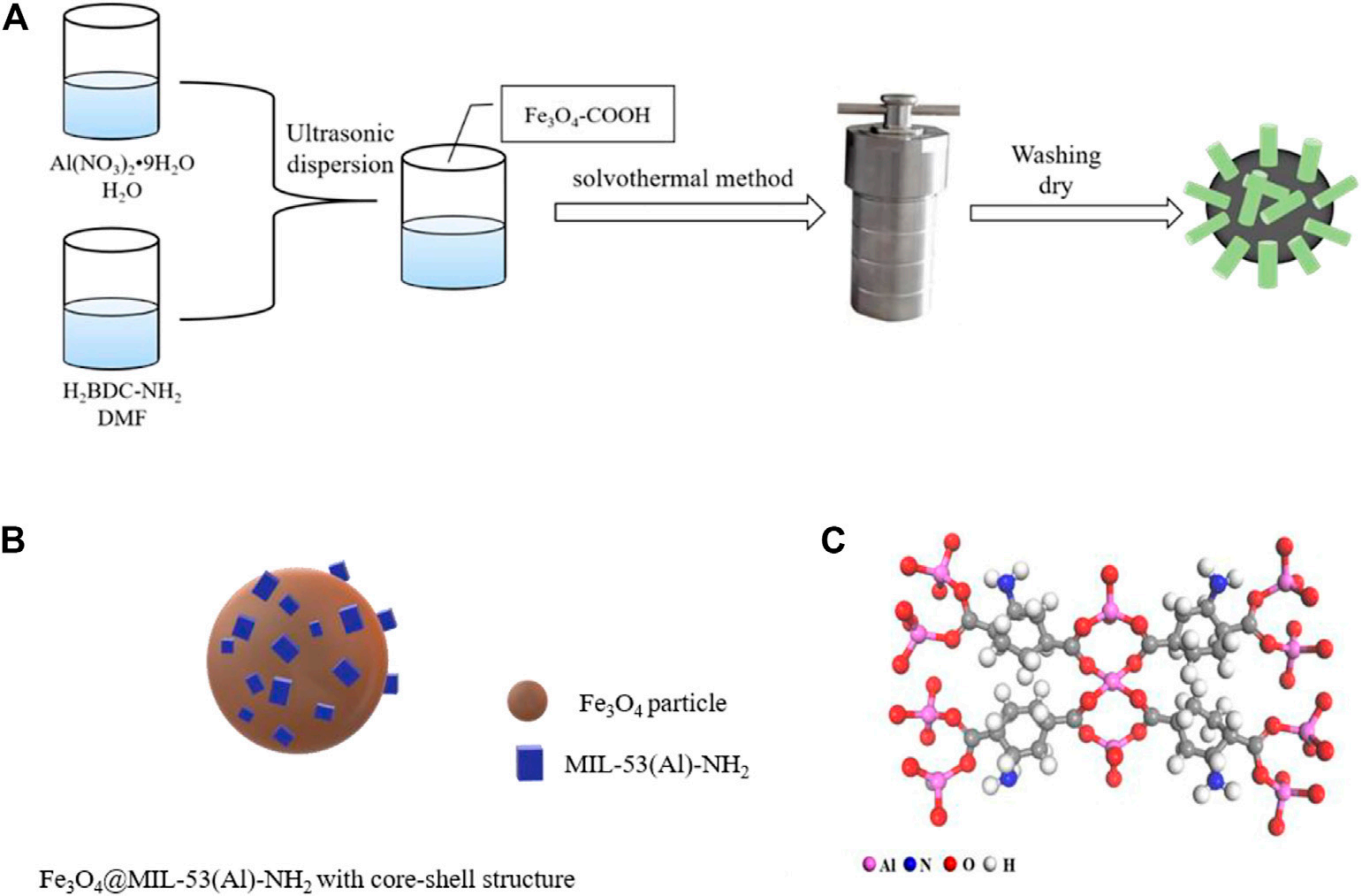
Illustration of **(A)** the Preparation Process of Fe_3_O_4_@MIL-53(Al)-NH_2_; **(B)** the core-shell structure of Fe_3_O_4_@MIL-53(Al)-NH_2_; **(C)** the chemical structure of MIL-53(Al)-NH_2_.

Scheme 1 illustrates the preparation of carboxylated Fe_3_O_4_-COOH particles. FeCl_3_·6H_2_O (1.3 g, 4.8 mmol) and trisodium citrate dihydrate (0.5 g, 1.7 mmol) were dissolved in 40 ml of ethylene glycol by ultrasonic stirring for 10 min. Sodium acetate (2.6 g, 31.7 mmol) was added, and ultrasound was performed for 30 min. The solution was then transferred to the polytetrafluoroethylene reactor and reacted at 200°C for 8 h. The solution was then cooled to ambient temperature. The prepared materials were magnetically separated using an external magnetic field, washed alternately with deionized water and ethanol, and dried at 60°C. Carboxylated Fe_3_O_4_-COOH particles were thus obtained.

Fe_3_O_4_@MIL-53(Al)-NH_2_ was fabricated using the following procedure: Aluminum nitrate nonahydrate (0.5625 g, 1.5 mmol) was dissolved in 10 ml of deionized water, and 2-aminoterephthalic acid (0.28 g, 1.5 mmol) was dissolved in 40 ml of DMF. After the two solutions were completely dissolved, they underwent ultrasonic mixing for 30 min Fe_3_O_4_-COOH (0.1 g, 0.4 mmol) was subsequently incorporated into the solution. The particles were evenly distributed by ultrasonic dispersion for another period of 10 min. The above-mentioned solution was then transferred to the Polytetrafluoroethylene reactor and reacted at 150°C for 24 h. At the end of the reaction, the solution was activated with DMF for 8 h under 130°C and then magnetized for magnetic separation. The solid obtained by magnetic separation was washed with methanol and stored at 80°C for 12 h to dye. Fe_3_O_4_@MIL-53(Al)-NH_2_ was ultimately obtained.

### Adsorption Experiments

In the adsorption experiments, BPA and TC were dissolved in deionized water to prepare different concentrations (20–150 mg/L). The adsorbents were added into 20 ml of a 100 mg/L dye solution, shaken at 500 rpm in a constant temperature oscillator for a given time. Ionic strength was modulated by adding sodium chloride with different masses. The pH values (3.0–11.0) were adjusted using 0.1 mol/L HCl and 0.1 mol/L NaOH solutions. The supernatant liquid was measured by UV−vis spectrophotometry. All parallel experiments were performed in triplicate to ensure accuracy, and the average results were employed for further data analysis. The removal efficiency of dyes and the adsorbed amount were calculated with the following formula:

$$dye\;removal\;efficiency\left(\%\right)=\frac{M_{0}-M}{M_{0}}$$ (1)

$$Q_{e}=\frac{\left(C_{0}-C_{e}\right)V}{m}$$ (2)

where *M*_0_ (g) is the initial mass of the dye, and *M* (g) is the mass of the dye after adsorption; *Q*_e_ (mg/g) is the adsorbed amount by the adsorbent at the adsorption equilibrium; *V* (ml) is the volume of the dye solution; and *m* (mg) is the adsorbent quantity.

Based on adsorption experiments, we discuss the adsorption kinetics models, adsorption isotherm and adsorption thermodynamics to explore the possible adsorption mechanism between adsorbent and dyes. Detailed calculation process was listed in the Supplementary Material.

### Regeneration Experiments

After each adsorption process, Fe_3_O_4_@MIL-53(Al)-NH_2_ was eluted with 0.1 mol/L NaOH and ethanol solution alternately until no dyes could be detected in the supernatant liquid. The regenerated Fe_3_O_4_@MIL-53(Al)-NH_2_ was dried overnight in a vacuum oven at 60°C.

## Results and Discussion

### Characterization of Fe_3_O_4_@MIL-53(Al)-NH_2_

**FIGURE 1:**
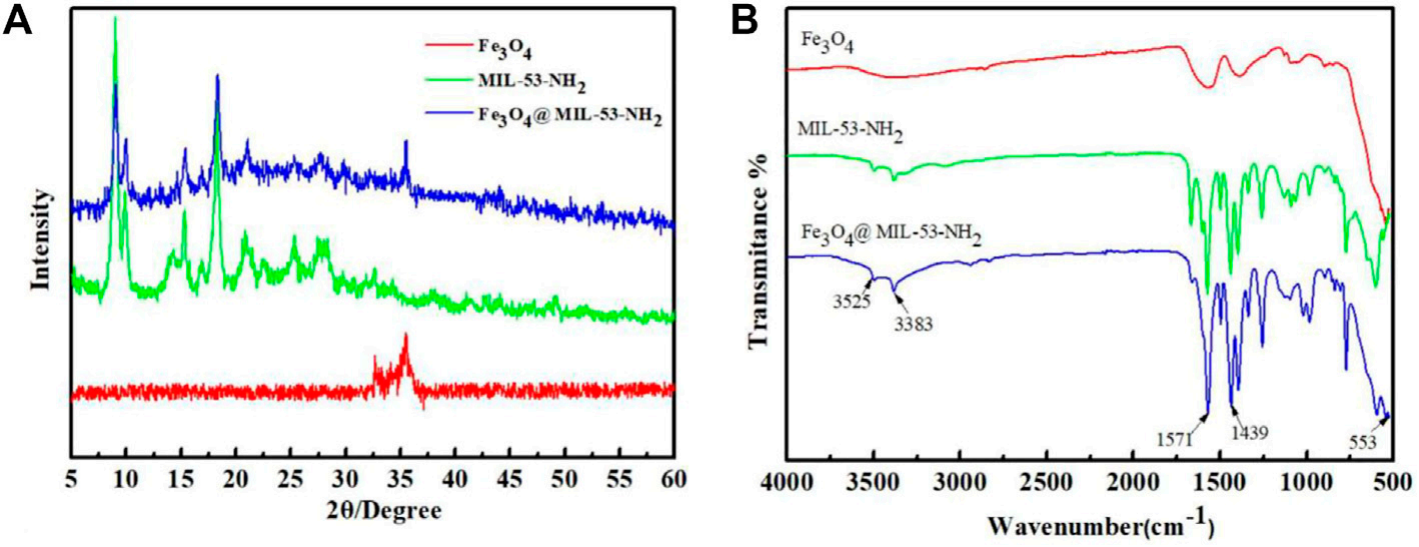
**(A)** XRD pattern of Fe_3_O_4_-COOH, MIL-53(Al)-NH_2_ and Fe_3_O_4_@MIL-53(Al)-NH_2_; **(B)** FT-IR spectra of Fe_3_O_4_-COOH, MIL-53(Al)-NH_2_ and Fe_3_O_4_@MIL-53(Al)-NH_2_.

Figure 1A presents the XRD patterns of Fe_3_O_4_-COOH, MIL-53(Al)-NH_2_, and Fe_3_O_4_@MIL-53(Al)-NH_2_. Both Fe_3_O_4_-COOH and Fe_3_O_4_@MIL-53(Al)-NH_2_ showed characteristic diffraction peaks of Fe at 35.5°, indicating the existence of Fe inFe_3_O_4_@MIL-53(Al)-NH_2_. Comparison of the MIL-53(Al)-NH_2_ spectra with Fe_3_O_4_@MIL-53(Al)-NH_2_ spectra, showed that both peaked at 8.9°, 10.15°, 15.3°, 18.38°, and 25.5°. This observation, was consistent with the literature (Qian et al., 2013), indicating that MIL-53(Al)-NH_2_ existed in the Fe_3_O_4_@MIL-53(Al)-NH_2_ composites, and the introduction of amino functional groups did not change the crystal type of the composite particles. That is, the XRD pattern of the composite particle Fe_3_O_4_@MIL-53(Al)-NH_2_ not only contains the characteristic peak of Fe_3_O_4_, but also retains the crystal diffraction peak of MIL-53(Al)-NH_2_, proving that the composite particle Fe_3_O_4_@MIL-53(Al)-NH_2_ was successfully prepared.

The FT-IR spectra of Fe_3_O_4_-COOH, MIL-53(Al)-NH_2_, and Fe_3_O_4_@MIL-53(Al)-NH_2_ are presented in Figure 1B. Both Fe_3_O_4_-COOH and Fe_3_O_4_@MIL-53(Al)-NH_2_ showed adsorption peaks at 553 cm^−1^, which was attributable to the presence of a Fe-O vibrational bond. Compared with those of pure Fe_3_O_4_ particles, the adsorption peaks of Fe_3_O_4_@MIL-53(Al)-NH_2_ appeared at 1571, 1439, and 1337 cm^−1^ because of the addition of MIL-53(Al)-NH_2_. The adsorption peak of 1000–1100 cm^−1^ is associated with Al-O, the adsorption peaks of 1596 and 1510 cm^−1^ are ascribed to the asymmetric stretching vibration of -CO, and the adsorption peak of 1690 cm^−1^ is attributed to the stretching vibration of -C=O, all of which are characteristic adsorption peaks of MIL-53(Al)-NH_2_.However, the adsorption peaks at 3,383 and 3,525 cm^−1^ verified the existence of amino functional groups in Fe_3_O_4_@MIL-53(Al)-NH_2_ due to the stretching vibration of -NH_2_.

**FIGURE 2:**
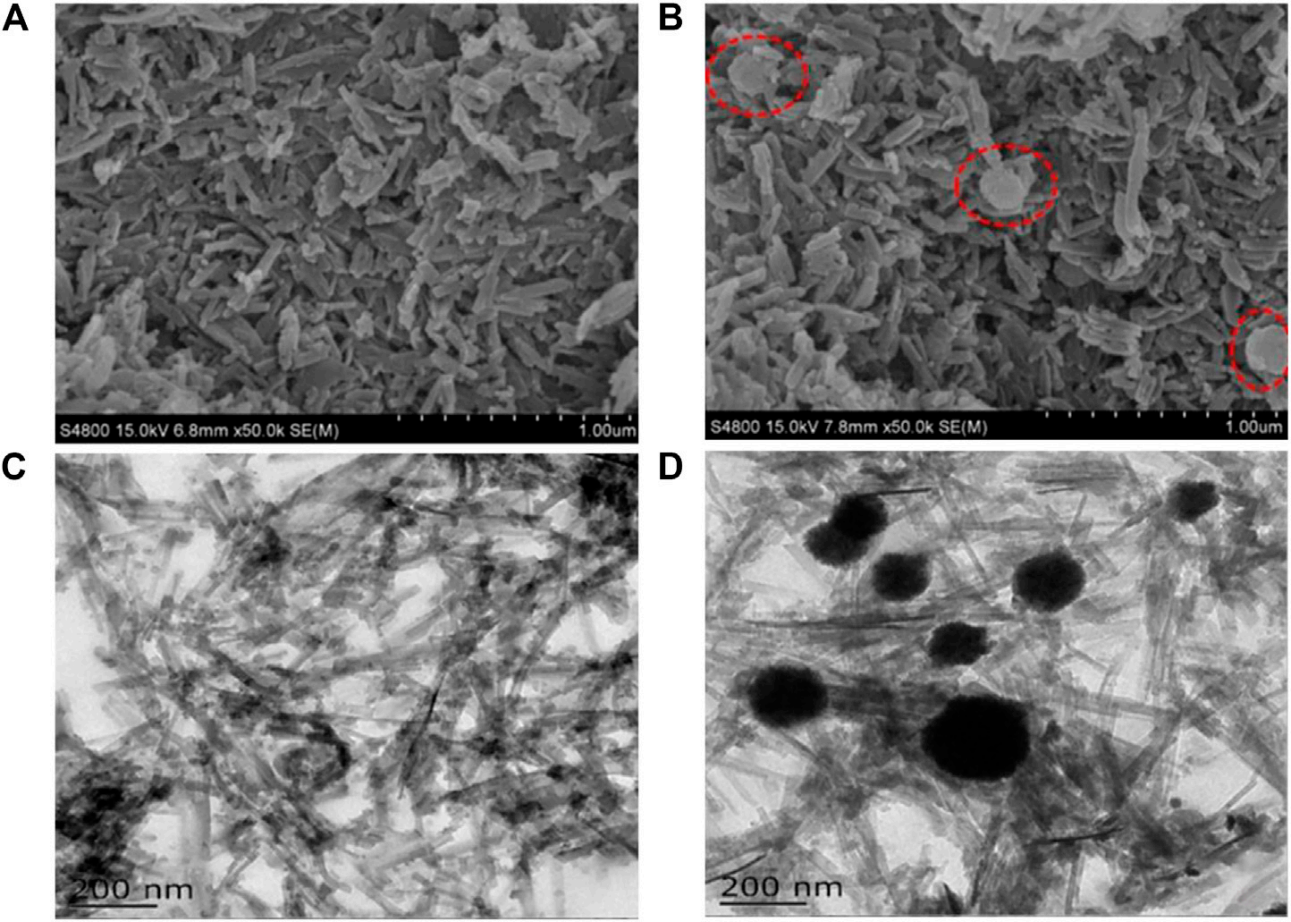
SEM images of **(A)** MIL-53(Al)-NH_2_ and **(B)** Fe_3_O_4_@MIL-53(Al)-NH_2_; TEM images of **(C)** MIL-53(Al)-NH_2_ and **(D)** Fe_3_O_4_@MIL-53(Al)-NH_2_.

The morphological characteristics, structures, and sizes of MIL-53(Al)-NH_2_ and Fe_3_O_4_@MIL-53(Al)-NH_2_ are shown in Figure 2. The SEM of MIL-53(Al)-NH_2_ structures was stacked on top of one another in a short stick shape before Fe_3_O_4_ was added. The SEM figure of Fe_3_O_4_@MIL-53(Al)-NH_2_ after the addition of Fe_3_O_4_ particles not only reveals the short rod-shaped MIL-53(Al)-NH_2_ but also shows some spherical objects (the red-dotted line). TEM imaging revealed that MIL-53(Al)-NH_2_ has a needle-stick morphology. After the addition of Fe_3_O_4_ particles, MIL-53(Al)-NH_2_ was surrounded by a black spherical Fe_3_O_4_ circumference and formed a Fe_3_O_4_@MIL-53(Al)-NH_2_ binary complex structure. These spherical particles consisted of Fe_3_O_4_ particles that were not completely coated with MIL-53(Al)-NH_2_. These exposed Fe_3_O_4_ particles not only verified the existence of Fe_3_O_4_ in the Fe_3_O_4_@MIL-53(Al)-NH_2_ composite particles but also indicated that most Fe_3_O_4_ was completely coated with MIL-53(Al)-NH_2_.

**FIGURE 3:**
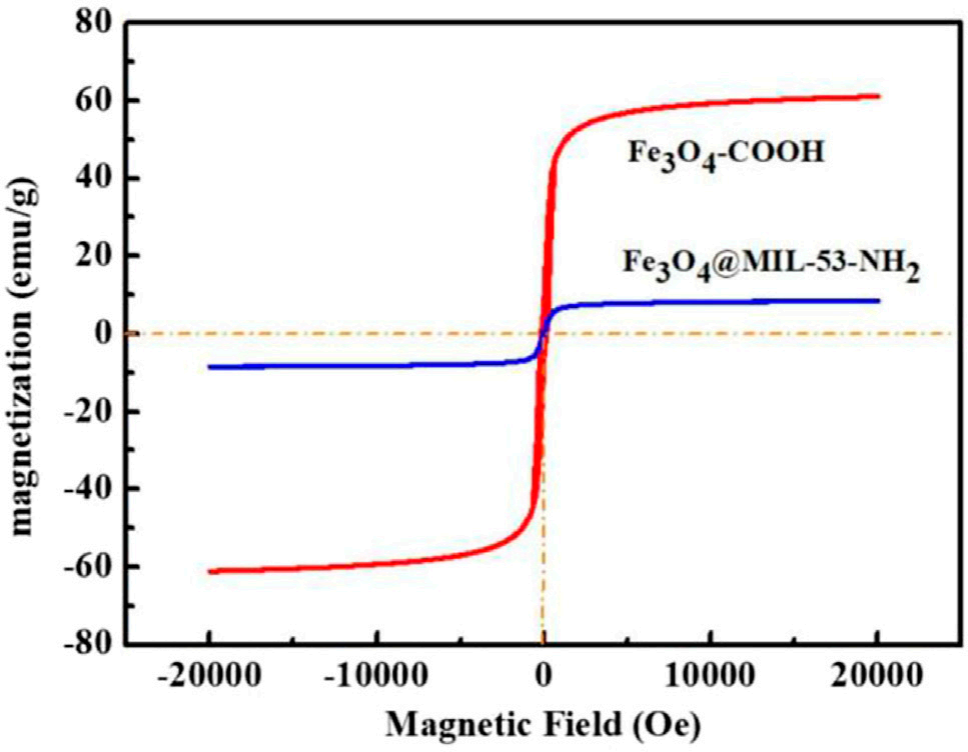
Vibrating sample magnetometry image of Fe_3_O_4_-COOH and Fe_3_O_4_@MIL-53(Al)-NH_2_.

The magnetic performances were observed using magnetic hysteresis loops with varying magnetic fields (Figure 3). The magnetic saturation strengths of Fe_3_O_4_-COOH and Fe_3_O_4_@MIL-53(Al)-NH_2_ were 61.118 and 10.529 emu/g, respectively. The magnetic saturation and intensity of Fe_3_O_4_@MIL-53(Al)-NH_2_ decreased after MIL-53(Al)-NH_2_ was added. The reason was that the addition of large amounts of MIL-53(Al)-NH_2_, which is a non-magnetic material, weakened the magnetic properties of the prepared samples. However, Fe_3_O_4_@MIL-53(Al)-NH_2_ composite particles could still undergo rapid separation under an external magnetic field, which is conducive to recycling.

**FIGURE 4:**
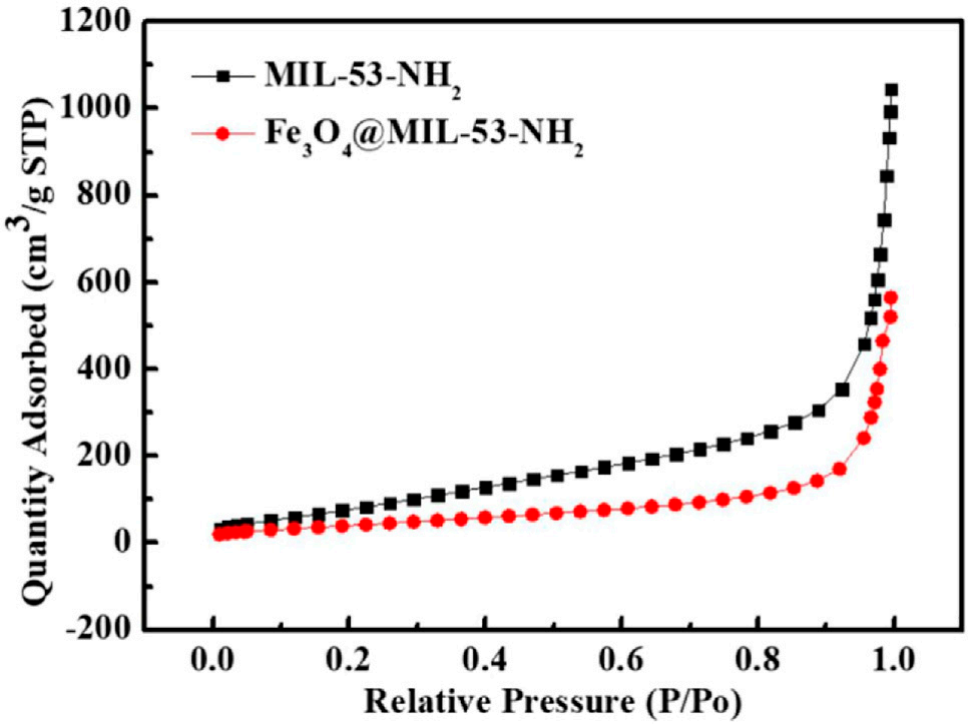
BET analysis of MIL-53(Al)-NH_2_ and Fe_3_O_4_@MIL-53(Al)-NH_2_

To study the changes in the specific surface area and micropore properties of the obtained materials before and after the addition of Fe_3_O_4_ particles, the nitrogen adsorption-desorption isothermal curves of MIL-53(Al)-NH_2_ and Fe_3_O_4_@MIL-53(Al)-NH_2_ were evaluated at 77 K (Figure 4). The specific surface area, total pore volume, and pore size of MIL-53(Al)-NH_2_ and Fe_3_O_4_@MIL-53(Al)-NH_2_ are listed in Table 1. As shown in Table 1, the specific surface area of MIL-53(Al)-NH_2_ is 317.3 m^2^/g and the total pore volume is 1.505 cm^2^/g. After the addition of Fe_3_O_4_ particles, the Fe_3_O_4_@MIL-53(Al)-NH_2_ composite particles were prepared, with a specific surface area of 151.9 m^2^/g and a total pore volume of 0.796 cm^2^/g. With the addition of Fe_3_O_4_, the material decreased in specific surface area and pore volume. The reason was that part of the non-porous Fe_3_O_4_ particles formed the pore structures of MIL-53(Al)-NH_2_ during preparation process. This process might have blocked some pores of the MOF material, reducing the specific surface area and pore volume. Similar occurrences were found in other MOF composites, such as SiO_2_ and MOF composites(Han et al., 2015; Han et al., 2016).

**TABLE 1:** BET data of MIL-53(Al)-NH_2_ and Fe_3_O_4_@MIL-53(Al)-NH_2_.

	Specific surface area (m^2^/g)	Pore volume (cm^2^/g)	Pore Size (cm^2^/g)
MIL-53-NH_2_	317.3	1.51	16.90
Fe_3_O_4_@MIL-53-NH_2_	151.9	0.80	18.95

### Adsorption of BPA and TC

#### Influence of the Amount of Adsorbent

**FIGURE 5:**
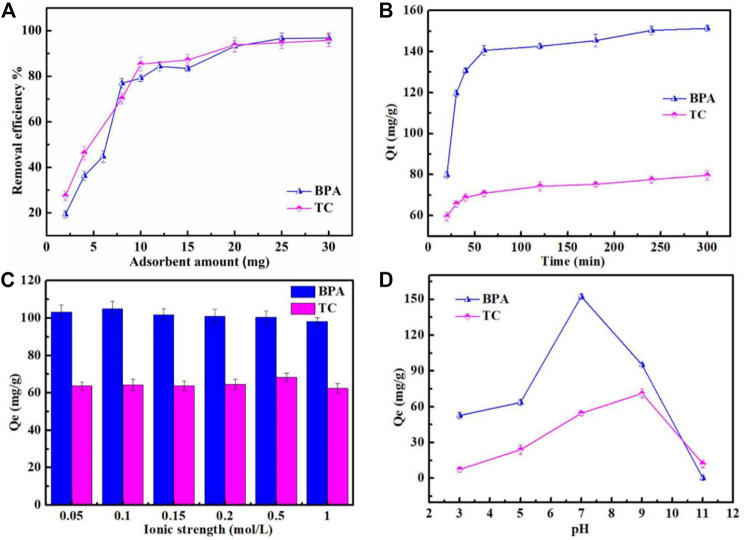
**(A)** influence of adsorbent amounts on BPA and TC; **(B)** influence of time on BPA and TC; and **(C)** influence of ionic strength on BPA and TC; **(D)** Influence of pH on BPA and TC.

The removal rates of BPA and tetracycline, increased with an increase in the amount of the Fe_3_O_4_@MIL-53(Al)-NH_2_ adsorbent (Figure 5A). The removal rates of BPA and tetracycline exceeded 80% when 10 mg of the adsorbent was added; with an increase in the amount of the adsorbent added, the two target pollutants showed no significant increases in the removal rate. The removal rates of both BPA and tetracycline exceeded 99 % when 30 mg of the adsorbent was added, and the pollutants were almost completely removed, indicating that Fe_3_O_4_@MIL-53(Al)-NH_2_ exerted a superior removal effect on the two pollutants. Therefore, in the subsequent experiments, the amount of adsorbent used for BPA and tetracycline removal was 0.5 g/L.

#### Influence of Adsorption Time

The effect of adsorption time from 20 to 300 min was evaluated. As shown in Figure 5B, as the adsorption time is extended, the adsorption capacities of BPA and tetracycline increase. When the adsorption time ranged from 20 to 60 min, the adsorption capacities largely increased at the 20–60 min time points. After 60 min, the adsorption capacity for BPA and tetracycline showed no significant change; thus, the adsorption equilibrium time of BPA and tetracycline was 60 min.

#### Influence of Ionic Strength

The influence of ionic strength as an important factor affecting the adsorption efficiency was determined by varying the NaCl concentration from 0.01 mol/L to 1.0 mol/L. As shown in Figure 5C, the amounts of BPA and tetracycline adsorbed do not significantly change with an increase in NaCl concentration. In this adsorption system, ion intensity only slightly affected the adsorption of BPA and tetracycline by Fe_3_O_4_@MIL-53(Al)-NH_2_. The reason is that in this system, the positive effect of salting-out and the competitive effect of salt ions cancel each other, or both effects were too weak to influence adsorption. Consequently, the effect of ionic strength on adsorption was insignificant, hence the absence of a significant change in the amounts of BPA and tetracycline adsorbed by Fe_3_O_4_@MIL-53(Al)-NH_2_ as ionic strength changes.

#### Influence of pH

The pH in the solution could exert a considerable influence on the adsorption of dyes. In this study, the influence of pH level, from 3 to 11, on the removal efficiency of the sample was measured (Figure 5D). The adsorption capacity of the adsorbent for BPA was smaller under acidic and alkaline conditions than that under neutral conditions. Particularly at pH = 11, the adsorption capacity of Fe_3_O_4_@MIL-53(Al)-NH_2_ for BPA was extremely low, with almost no adsorption. The reason is that when pH is greater than 9, BPA ionized to form the monovalent anion HBPA^−^ and the bivalent anion BPA^2−^; moreover, BPA anions and the negatively charged adsorbent surface produce electrostatic repulsion, resulting in reduced adsorption. When the solution is acidic, the abundant H^+^ in the solution and the positively charged adsorbent on the surface also repel each other. Therefore, BPA is most highly adsorbed under neutral conditions (Li et al., 2015).

The adsorption capacity for tetracycline increased with an increase in pH, and reached the maximum at pH = 9. Under highly acidic (pH = 3) and highly basic (pH = 11) conditions, the adsorption capacity for tetracycline was low, mainly owing to charge repulsion between the adsorbent and the adsorbate. At pH = 3, tetracycline existed in an aqueous solution in the form of cationic TC^+^, which generated electrostatic repulsion with the adsorbent exhibiting a positive surface charge. At pH = 11, tetracycline was ionized, forming anion TC^2−^ and producing charge repulsion with the adsorbent exhibiting a negative surface charge. At pH = 9, the negatively charged tetracycline ion and the negatively charged adsorbent were expected to exhibit electrostatic repulsion, resulting in low adsorption; however, this expected outcome was contrary to the experimental results. This inconsistency indicates that the primary interaction between the adsorbent and the adsorbate is not a charge–charge interaction but instead, but H bond and п-п conjugation(Xiaonuo et al., 2018; Yang et al., 2019). In conclusion, optimal adsorption was achieved at pH = 7 for BPA and pH = 9 for tetracycline.

### Adsorption Kinetics

Adsorption kinetics is used to elucidate adsorption behaviors, including mass transfer, chemical reaction, and determining the rate of adsorption. In this section, the pseudo-first-order, pseudo-second-order, and intraparticle diffusion models are reviewed to research the experimental data.

**FIGURE 6:**
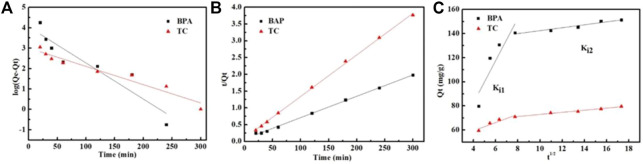
Fit of kinetic data to **(A)** the pseudo-first-order model, **(B)** the pseudo-second-order model, and **(C)** the intraparticle diffusion model on BPA and TC.

The linear regression curves of the three models for BPA and TC adsorption are presented in Figures 6A–C, and supporting date are given in Table 2. The pseudo-first-order kinetic fitting data for Fe_3_O_4_@MIL-53(Al)-NH_2_ adsorption of BPA and tetracycline were poor, and the fitting correlation coefficient *R* ^2^ was low. Moreover, the adsorption capacity at equilibrium, calculated using quasi-first-order kinetics, largely varied from that determined by experimental testing. Thus, the quasi-first-order kinetics is not suitable for describing the adsorption of BPA and tetracycline on Fe_3_O_4_@MIL-53(Al)-NH_2_. The quasi-second-order kinetics was used to fit Fe_3_O_4_@MIL-53(Al)-NH_2_ adsorption of BPA and tetracycline, and the *R* ^2^ coefficients were 0.9984 and 0.9992 respectively, showing a superior fitting effect. In addition, the amount adsorbed at equilibrium, as determined from the fitting calculation, was slightly different from that determined based on the experiment. Therefore, the quasi-second-order kinetic model could better describe the adsorption the adsorption of BPA and tetracycline on Fe_3_O_4_@MIL-53(Al)-NH_2_; that is, chemical adsorption occurred in both pollutant adsorption systems.

**TABLE 2:** Parameters of pseudo-first-order, pseudo-second-order, and intraparticle diffusion models.

Kinetics	Parameters	dyes	
		BPA	TC
	*Q*_cal_^a^ (mg/g)	158.7	83.3
Pseudo-second-order	*Q*_exp_^b^ (mg/g)	150.9	80.7
	*k*_2_ (g/mg/min)	5.4 × 10^–4^	1.4 × 10^–3^
	*R* ^2^	0.9984	0.9992
	*Q*_cal_^a^ (mg/g)	57.0	20.2
Pseudo-First-Order	*k*_1_ (min^−1^)	0.0177	0.0080
	*R* ^2^	0.8502	0.9344
	*k*_i1_ (m/g^/^min)	17.55	3.40
	*C*_1_ (mg/g)	12.3	45.8
	*R* ^2^	0.7476	0.8493
Intraparticle Diffusion Models	*k*_i2_ (mg/g^/^min^1/2^)	1.22	0.87
	*C*_2_ (mg/g)	130.3	64.2
	*R* ^2^	0.9259	0.9755

a *Q*_e,exp_ is the equilibrium adsorption capacities according to the experimental results.

b *Q*_e,cal_ is determined by the linear fitting from the kinetic models.

The fitting curve of the intramolecular diffusion model consisted of several straight lines, none of which passed through the origin, indicating that adsorption is affected by multiple diffusion processes and not solely determined by internal diffusion steps. Moreover, the fitting coefficient of the second stage is higher than that of the first stage, indicating that the diffusion of pollutant molecules on the adsorbent pore is the main control step in the adsorption process. Therefore, the adsorption of BPA and tetracycline on Fe_3_O_4_@MIL-53(Al)-NH_2_ was conformed more to the quasi-second-order kinetics, according to kinetic analysis. Moreover, the adsorption process was influenced by various diffusion processes, and intramolecular diffusion was the controlling step.

### Adsorption Isotherms

**FIGURE 7:**
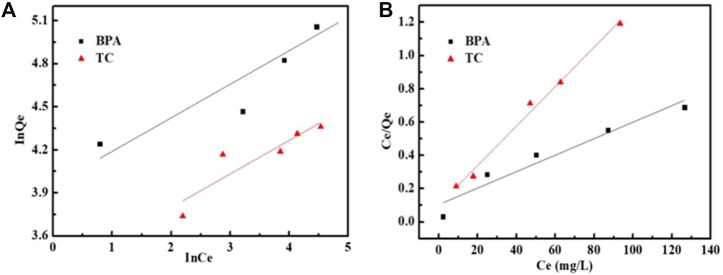
Adsorption isotherm curves of BPA and TC on Fe_3_O_4_@MIL-53(Al)-NH_2_. **(A)** Freundlich model; **(B)** Langmuir model.

The adsorption isotherm is important to determine the adsorption type and strength between the adsorbents and the target compounds in an adsorption batch system. As shown in Figure 7 and Table 3, the Freundlich model has a poor fitting effect and a poor fitting coefficient. These results indicated that the Freundlich isotherm model was not suitable for describing the adsorption mechanism of Fe_3_O_4_@MIL-53(Al)-NH_2_ for these two pollutants. However, the Langmuir model fitting showed a higher *R* ^2^ coefficient of Fe_3_O_4_@MIL-53(Al)-NH_2_ adsorption for BPA and tetracycline removal, indicating that the adsorption process was more consistent with the Langmuir model and exhibited a single-layer homogeneous adsorption. Moreover, the maximum adsorption capacity of Fe_3_O_4_@MIL-53(Al)-NH_2_ for BPA was 234.1 mg/g, and that for tetracycline was 84.8 mg/g.

**TABLE 3:** Isotherm parameters of Fe_3_O_4_@MIL-53(Al)-NH_2_ for BPA and TC adsorption.

	Langmuir model			Freundlich model		
Dyes	*Q*_max_ (mg/g)	*K*_L_ (L/mg)	*R* ^2^	1/*n*	*K*_F_	*R* ^2^
BPA	234.1	0.047	0.9226	0.2339	52.20	0.8200
TC	84.8	0.116	0.9909	0.2334	27.99	0.7734

The comparison of the adsorbent with developed in this work with previously-reported conventional adsorbents for the removal of BPA and TC is summarized in Table 4. Fe_3_O_4_@MIL-53(Al)-NH_2_ has a higher adsorption capacity than other adsorbents with the assistance of amido-functionalization and can be easily separated by a magnetic field, allowing it to act as a potential purifying agent for removing cationic dyes from wastewater.

**TABLE 4:** Comparison of BPA and TC Adsorption Capacity of Fe_3_O_4_@MIL-53(Al)-NH_2_ with Other Adsorbents (mg g^−1^).

dyes	Adsorbent	*Q*_max_ (mg/g)	dyes	Adsorbent	*Q*_max_ (mg/g)
BPA	Granular activated carbon (Kim et al., 2015)	118.0 (298 K)	TC	Kaolinite (Li et al., 2010)	4.3(295 K)
	Graphene(Xu et al., 2012)	181.8 (302 K)		Activated sludge (Prado et al., 2009)	72.0 (N.A.)
	mesostructured MIL-53(Al) (Zhou et al., 2013)	465.0 (273 K)		MIL-53(Fe) (Yu et al., 2019)	248.3 (298 K)
	Fe_3_O_4_@Dex(Icten and Ozer, 2021)	66.2 (297 K)		NH_2_-MIL-53(Fe) (Yu et al., 2019)	265.3 (298 K)
	MOF:Fe_3_O_4_@Dex(Icten and Ozer, 2021)	374.0 (297 K)		CuCo/MIL-101 (Jin et al., 2019)	59.9 (298 K)
	MIL-53(Al)@SiO_2_ (Boontongto and Burakham, 2020)	134.7 (303 K)		MIL-g-0.3 (Zheng et al., 2020)	154.5 (298 K)
	Fe_3_O_4_@MIL-53(Al)-NH_2_	234.1 (298 K)		Fe_3_O_4_@MIL-53(Al)-NH_2_	84.8 (298 K)

### Adsorption Thermodynamics

To further explore the type of adsorption type Fe_3_O_4_@MIL-53(Al)-NH_2_ of bisphenol A (BPA) and tetracycline adsorption type, and explore the possible adsorption mechanism, the process of adsorption the change in enthalpy change (Δ*H*°), change in Gibbs free energy (Δ*G*°) and change in entropy (Δ*S*°) were calculated (calculation process in Supplementary Material). The results are listed in Table 5. As shown in Table 5, Δ*G*°<0, Δ*H*°>0, proving the adsorption of BPA and tetracycline on Fe_3_O_4_@MIL-53(Al)-NH_2_ is a spontaneous and endothermic process. The absolute value of Δ*G*° increases as temperature rises, indicating that high temperature contributes to the adsorption of these two pollutants. When the absolute value of Δ*H*° is less than 84 kJ/mol, the adsorption process was mainly a physical adsorption(Ren and Xiong, 2013). Therefore, the adsorption of BPA and tetracycline on Fe_3_O_4_@MIL-53(Al)-NH_2_ was mainly a physical adsorption process.

**TABLE 5:** Thermodynamic parameters for BPA and TC adsorption on Fe_3_O_4_@MIL-53(Al)-NH_2_.

Dyes	*T* (K)	Δ*G*° (J/mol)	Δ*H*° (kJ/mol)	Δ*S*° (J/mol/K)
	298	−2,092		
BPA	308	−3,415	37.31	132.2
	318	−4,737		
	298	−2,400		
TC	308	−3,626	34.14	122.6
	318	−4,852		

In thermodynamics, Δ*S*° represents the degree of chaos in the adsorption system. The adsorption of BPA and tetracycline on Fe_3_O_4_@MIL-53(Al)-NH_2_ showed that Δ*S*° was greater than 0. This result indicates that the adsorption of these two kinds of pollutants was a process of entropy in the adsorption system, with increasing disorder. The reason was that during the adsorption of BPA and tetracycline on Fe_3_O_4_@MIL-53(Al)-NH_2_, the desorption of water molecules on the surface of the adsorbent was greater than that of the pollutant molecules. The adsorption of BPA or tetracycline molecules required the desorption of more water molecules, leading to an increase in disorder in the solution.

### Reusability Studies

Excellent adsorbents exhibit fast adsorption and superior recycling efficiency. Therefore, the reusability of Fe_3_O_4_@MIL-53(Al)-NH_2_ was evaluated.

**FIGURE 8:**
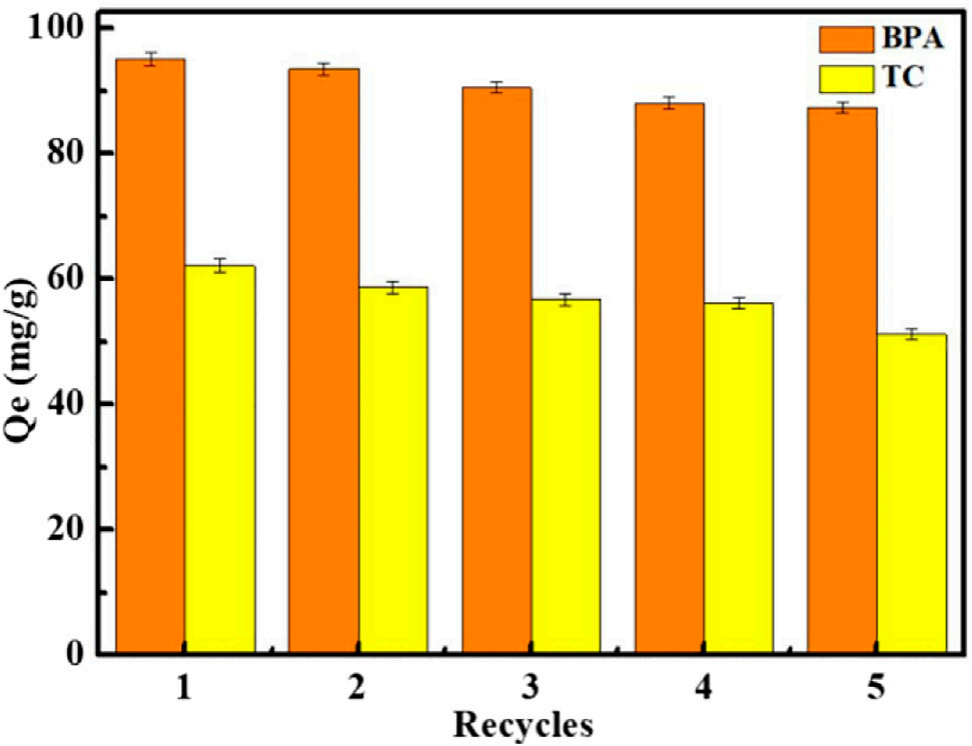
Qc (mg/g) of BPA and TC after five recycles.

After the adsorption of BPA or tetracycline was completed, Fe_3_O_4_@MIL-53(Al)-NH_2_ was eluted with a methanol solution several times and then placed in a drying oven for recycling. The adsorption effect after five cycles of regeneration is shown in Figure 8. After five times of desorption regeneration, the adsorption capacity for BPA exceeded 90 mg/g, and that for tetracycline exceeded above 50 mg/g; no significant decrease in adsorption capacity was found after multiple cycles. These experimental results prove that the prepared materials exhibit satisfactory reusability and broad application potential. This finding suggests that the adsorption of BPA and tetracycline on Fe_3_O_4_@MIL-53(Al)-NH_2_ is mainly physical adsorption process.

## Conclusion

In this study, an amido-functionalized MOFs adsorbent (Fe_3_O_4_@MIL-53(Al)-NH_2_) was prepared by solvothermal method synthesis for BPA and TC removal. The morphology and structure of Fe_3_O_4_@MIL-53(Al)-NH_2_ were well characterized by TEM, SEM, FT-IR, XRD, and VSM. The influence on the adsorption efficiency, adsorption capacity, adsorption time, ionic strength, and pH were explored to determine the optimized adsorption conditions. The maximum adsorption capacity of BPA was 234.1 mg/g, and that of TC was 84.8 mg/g, respectively, superior to traditional adsorbents and can be comparable to many reported MOF adsorbents for the removal of water contamination. The adsorption kinetics and equilibrium adsorption data indicated that the adsorption processes of BPA and TC were more compatible with the pseudo-second-order kinetic model and the Langmuir model, respectively. The thermodynamic values, including the enthalpy change (Δ*H*°), Gibbs free energy change (Δ*G*°), and entropy (Δ*S*°) show that the adsorption of the aforementioned contaminant was spontaneous and endothermic. Moreover, the Fe_3_O_4_@MIL-53(Al)-NH_2_ adsorbent exhibited good regeneration and reusability after five cyclic utilization, owing to the magnetism of Fe_3_O_4_. The high adsorption capacity and good recycling capability of Fe_3_O_4_@MIL-53(Al)-NH_2_ render it suitable as an adsorbent for future health and the ecological environment.

## Data Availability

The raw data supporting the conclusions of this article will be made available by the authors, without undue reservation.
